# 358. Surveillance of Activity of Antifungals: Data from the Analysis of Resistance in Antifungals (ARIA) 2020 Study

**DOI:** 10.1093/ofid/ofac492.436

**Published:** 2022-12-15

**Authors:** Stephen Hawser, Nimmi Kothari, Bárbara Lemos

**Affiliations:** IHMA Europe, Monthey, Valais, Switzerland; IHMA, Monthey, Valais, Switzerland; IHMA, Monthey, Valais, Switzerland

## Abstract

**Background:**

ARIA is a new annual global surveillance initiative collecting yeast and fungal isolates from worldwide designed to determine resistance to antifungal agents and trends over time. The ARIA program was developed in 2020 to provide a repository of recent clinical fungal isolates with known susceptibility profiles and to monitor resistance trends over time. ARIA reports the susceptibility patterns of its earliest data concerning echinocandins, second-generation triazoles, and fluconazole against clinical *Candida albicans*, non-albicans strains including *C. auris*, *Aspergillus* and *Fusarium* isolates from worldwide sources.

**Methods:**

Isolates were collected from hospital worldwide during 2020, shipped to a central laboratory and re-identified by MALDI-TOF or molecular methods. MIC tests were performed by broth microdilution method in line with CLSI susceptibility testing standards. Percentage of susceptibility was calculated according to CLSI breakpoints. Antifungals tested were amphotericin B (AMB), anidulafungin (ANID), fluconazole (FLU), isavuconazole (ISA), micafungin (MIC), posaconazole (POS), and voriconazole (VOR). All testing was performed according to CLSI M27-A4 and M38-A2 methodologies.

**Results:**

See table.

**Figure:**
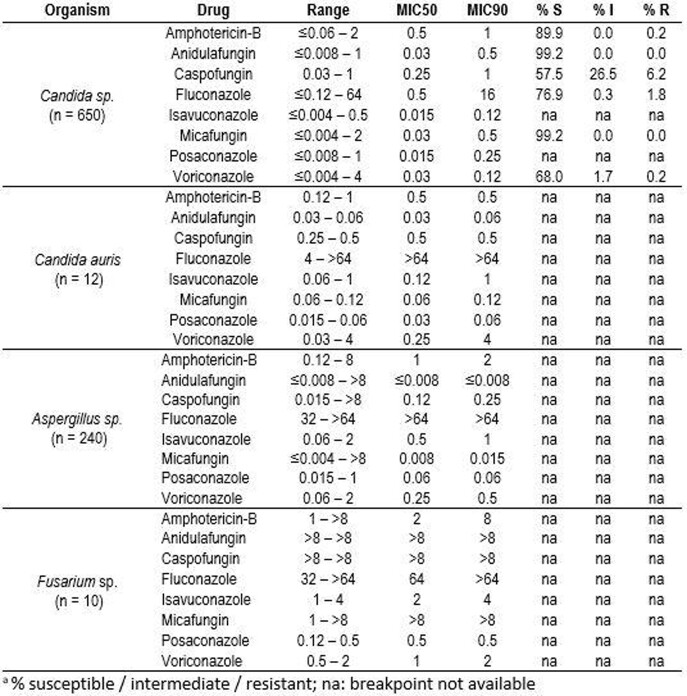

**Conclusion:**

Ongoing antifungal resistance surveillance like the ARIA program is of utmost importance in order to monitor the efficacy of traditional empirical therapy and for the development of novel antifungal agents. This repository and ongoing ARIA program will provide a resource to better support the biopharmaceutical industry’s goals to develop new and more potent antifungal agents.

**Disclosures:**

**All Authors**: No reported disclosures.

